# American Academy of Dental Science

**Published:** 1891-01

**Authors:** 

**Affiliations:** American Academy Dental Science


### AMERICAN ACADEMY OF DENTAL SCIENCE.[1]

The American Academy of Dental Science held its regular monthly meeting March 5, 1890, in the Boston Medical Library Association rooms, President Seabury in the chair.

*President Seabury.*—If there are no reports of committees, the next thing in order is Dr. Brackett's paper "Concerning Third Molars."

(For Dr. Brackett's paper, see page 10.)

*President Seabury.*—The matter is now open for discussion and general remarks.

*Dr. Chandler.*—This matter of the non-appearance of the wisdom tooth is, I think, very common, and getting to be more so every day. I have seen quite a number of instances among my patients where it had never appeared, and in many other cases there was a sort of a beany look to the tooth which seemed to indicate a want of development. I myself have had but three of my

---

[1] These proceedings have been delayed by the non-reception of the paper of Dr. Brackett, and are necessarily published out of the regular order.—[Ed.]

wisdom teeth, and those were extracted very shortly after appearing. It also seems to me that the lateral is disappearing in the same way. I have seen quite a number of instances of it, and I remember one patient where the upper six-year molars were extracted on both sides in the usual routine way practised almost universally some years ago. The lady never had the laterals, and the consequence was that the upper centrals shut inside of the under teeth. When she came to me she was about thirty years of age, a young lady in society, and quite sensitive as to the appearance of her teeth. I began a course of regulating, and succeeded in getting them to bite square, but I was never able to do anything more, as she gave it up there, being satisfied with what she had gained.

With reference to the rank of the wisdom tooth in the tables which Dr. Brackett has read from Magitot and Hitchcock, I think there is a fallacy in those tables that has not been noticed, and that is, that the wisdom teeth are twelve years younger than the first molars, and consequently have twelve years more chance. To reckon the comparative durability of the wisdom teeth without allowing for this advantage is not just. The canines come after the six-year molars, then the twelve-year molars, and the eighteen-year molars; and there are hundreds of first molars extracted before the third molars make their appearance. It is just so with the hair and the whiskers,—the color of the hair always turns first, and you will find it so universally; apparently it is the difference in the age.

*Dr. Fillebrown.*—Perhaps Dr. Brackett would like a little company for his cases of paralysis. Some six or eight years ago a lady, who had been a patient of mine for a long time, came to me with a left inferior third molar crowded, and with much inflammation in the parts around it. It seemed best to extract it. I experienced no difficulty in performing the operation, had no hesitancy in deciding that it was the best thing to do, and there was no reason to expect injury to result. A decided paralysis of the inferior maxillary nerve followed, and, with little improvement, has continued to this day. In the whole left mental region of the face sensation is considerably less than normal. There was also a little motor paralysis, which has almost disappeared, but the sensory paralysis remains. It is not serious, only uncomfortable, as Dr. Brackett remarked in regard to some of his patients. I can fully corroborate the statement of Dr. Brackett with regard to the irregularity which the third molar causes when there is lack of room. I have observed it in a number of cases where the teeth, before the eruption of the third molars, were entirely regular in front, evenly situated, and the cuspids properly in position.

When the third molars did appear, the incisors became irregular and remained so. I have seen that in four or five different cases, and it has seemed to me that if the third molar had been taken out a little sooner there would have been no trouble of that kind. This effect of the third molars is more marked in the lower jaw. I do feel that there is a tendency to an elimination of the third molar. I am not fully up on the doctrine of evolution. Its general principles I believe in, but whether a man is an ape or not I am not fully decided, though a good many of them act like it.

I don't know whether evolution would be able to take a tooth out of mankind, or whether we would have to wait until some higher order is developed from this lower order which we call man. One thing we know that, going back among animals, we find that a complete set of teeth numbered forty-four, and, as I understand it, there have been eliminated in all twelve teeth, leaving thirty-two for the animal man. I suspect that these eliminations have been controlled by necessity. A thing that is not needed is very apt to cease to exist. You let a race go on for generations and not use a hand or an arm, or any other part of the body, you would find the unused member growing smaller, and eventually it would cease to exist. In our higher civilized races of men the necessity for teeth is not so great as it was among the animals that had forty-four, so the tendency is to decrease the number. If the number of a complete set of teeth has been decreased by twelve down to thirty-two, I see no reason why it should not be still further decreased to twenty-eight, and I believe that that is the tendency.

I was interested in these cases of impacted third molars that Dr. Brackett mentioned. I have had several cases of this kind, but have very seldom found it necessary to remove the second molar; in fact, I remember of but one case where I thought it necessary. In several instances I have ground off the third molar, as Dr. Brackett has represented. In other cases I have ground it a little, impacted rubber between it and the second molar, and gradually moved them apart so as to raise the crown of the third molar by that of the second. I would say that I have generally used the Physick forceps for extracting the third molars; and usually it will elevate them from their position remarkably quick and easy. A while since I had a patient who needed to have an upper third molar extracted. Within two years two dentists (I

was one) had expended their full force upon the tooth, using the ordinary forceps, without being able to move it from its position. Subsequently I ventured to use the Physick forceps. It was the first time I had used it in the upper jaw, fearing the breaking of the tuberosity. It was one of the greatest successes of my life. The tooth was removed with ease, and there were no signs of fracture. The patient was a surgeon, and understood the difficulty of the operation, and we were both much pleased at the result.

*Dr. Meriam.*—There is such a judicial character about all of Dr. Brackett's work that we can hardly hope to improve upon it. One case that I have had, and which I have spoken of here before, was very much worse than anything which he has mentioned. It was a case of impacted third molar, where the tooth was inclined so much that the distal surface was uppermost, and doing duty as a grinding surface, and the anterior cusps were locked behind the sound molar beneath the gum. I did what I have never heard of being done before,—drilled into the tooth far enough to employ arsenic to destroy the pulp, then with a hard rubber and corundum disk made a cut just in front of the distal cusps as far down as possible, then with a fissure bur, with end sharpened like a drill, drilled through to the membranes beneath. I drilled standing at the back of the chair and drawing the cheek back as much as possible. I followed the first drilling with a safe-end fissure bur of the right size, and, cutting laterally, soon removed a section representing fully a third of the crown; the tooth was then removed without difficulty, greatly to the satisfaction of my patient, for the tooth had distressed him very much, and its extraction had been dreaded both by himself and practitioners who had seen it. I include myself especially in the timid ones. But one morning the spirit moved me, and I "was bold." We can, I think, save much suffering by first destroying the pulps of teeth we are to grind or divide, previous to extraction.

*Dr. Briggs.*—It would seem that any one, after listening to Dr. Brackett's admirable paper, would have to be possessed of considerable temerity to announce a different method, but I have had cases of impacted third molars where I have extracted the second molar and produced good results. I have a very high opinion of the third molar, as high as of any tooth in the mouth, and I think that its faults are due to its environment,—that it is more sinned against than sinning, and that placed as far forward in the mouth as another tooth, it is as good a tooth as we have, but being placed as it is, with the cheek lying upon it, thereby rendering it impossible for the brush to reach it and keep it clean, it of course decays rapidly and becomes troublesome. In those cases, I think, oftentimes the third molar should be taken out, and I do extract them.

In cases where they erupt almost completely, but do not come up entirely, because they meet the upper molar and the bite in the angle of the jaw is so short, I have seen considerable suppuration around the tooth which I have supposed was due to a pocket between the gum and the tooth caused by the non-attachment of gum to the enamel. In some cases I have cured it by grinding an upper or lower molar until it could erupt further. When it is advisable to make more room to prevent the third molar from crowding, I have had cases in which I have taken out the second molar. In those cases of the third molar erupting a very small portion of its crown accompanied with a great deal of pain of the neuralgic sort, spreading about the jaws and the head, I have extracted the second molar, and in all the cases that I have so done, the neuralgia has been cured, and the third molar has swung into line perfectly, and has been an excellent tooth, as good, if not better, than the second molar, and I have also found in most of those cases that the second molar was more or less injured by the impact. It had become absorbed at the point of contact, hollowing in so as to make a shoulder,—a place away down out of the way,—which would, in the future, produce a cavity capable of quickly reaching the pulp. This fact has added to the causes of my congratulation at having taken out the second molar.

*Dr. Fillebrown.*—I can keep Dr. Briggs company in his view of the matter. One case presented itself to me not long ago in which I extracted an impacted third molar with great difficulty. I had to grind away the tooth and use rubber to separate, and it took a long time before I could get at the tooth to extract it, when, lo and behold! the distal surface of the second molar was a nest of decay. If I had taken out the second molar, it would have been a great deal better for the patient and have saved us both considerable time and trouble.

*Dr. Andrews.*—I recall one or two instances in which the extraction of a third molar gave me a good deal of trouble. Twenty years ago I had a case in which I broke the points of two pairs of forceps and spent nearly two hours in trying to extract a wisdom tooth,—and strange to say the gentleman is still a patient of mine. The tooth inclined forward, and was butting against the twelve-year molar, and was entirely under the gum.

I tried quite a while to get hold of the tooth, but the instrument would slip. Finally, with the engine, I drilled a couple of holes to hold the points of the cow-horn forceps, and was able to extract the tooth. This method is not original with me; somebody told me of it. In this case it worked admirably.

*Dr. Williams.*—I was very glad to hear in Dr. Brackett's excellent paper that he does not absolutely condemn the wisdom tooth. Patients often say, when I have spoken of filling a cavity in a wisdom tooth, Is it not better to throw it away? But I tell them I have seen wisdom teeth outlast all the others, and sometimes become valuable supports in retaining artificial sets. There are several things about them in which they differ from other teeth, and one thing that I have noticed is that the dentine is apt to be more sensitive than the average of dentine of the other molars, in fact, nearly resembling pulp sensitiveness. My attention was first called to it through an experience of my own. I had considerable trouble with an upper left wisdom tooth, and, like many of our patients, I bore it for a week or two before I decided to have it attended to. I thought, from the sensation, that it was the result of pulp exposure, and made a statement to that effect to the dentist to whom I applied. He, having a slight opinion of wisdom teeth, said that it had better come out, and out it came. On glancing at it, we found that simply the enamel was decayed through. It was the dentine which had given me all the trouble, and I have since found in wisdom teeth that the sensation of the dentine often is very similar to the sensation of an exposed pulp, therefore I make that allowance always.

As Dr. Briggs has said, the wisdom teeth are sometimes the cause of general neuralgia. I remember a patient, a lady, who had been treated six months for neuralgia, and she repeatedly suggested that the teeth might have something to do with it, and finally decided to have them extracted. She came to me, and I saw that they were very important teeth for chewing, but the pulps were exposed. I told her that very possibly her neuralgia came from them, and that if she had plenty of teeth she might be able to do without the troublesome ones, but that, under the circumstances, I thought it would be better to fill and save them. She consented, and those teeth lasted her for years. The physician had to stop his treatment, as the case ended as far as it required medical treatment. I do not know but that they are still in her mouth.

In regard to those cases where the wisdom teeth impinge against the second molar I have had some very complicated cases and, of course, have had my experience in trying to extract them. I, too, have tried the grinding process. In some cases, by a very slight grinding with a diamond disk, I have obtained just the chance, if there was no chance before, to pack some cotton between the second and third molars, then I have put a waxed tape between them, after that perhaps increased the folds, so that the tooth as it grew and pressed against the other tooth would have a chance to slide up. In several cases there has been success in this way.

*Dr. Chandler.*—Speaking of the evolution of the wisdom teeth out of the jaw, I have one case where it seems to be evolving the other way. The patient is a man fully fifty-five years of age, and he has six wisdom teeth, two of course being supernumeraries. Four of them are in the lower jaw, and they are all perfectly formed and in good condition, and he has a very good set of teeth otherwise, and a pretty large jaw. There they stand and are likely to stand until he dies. It is the only case of the sort that I have ever had in my practice.

*Dr. Andrews.*—I spoke of a case of that kind at our meeting in Cambridge, in which there were four extra teeth, making eight wisdom teeth in one mouth. I gave the name of the patient—a lady residing in Tiverton, R. I.—to Dr. Brackett, thinking he might have an opportunity of seeing this remarkable case.

*President Seabury.*—Since our last meeting I extracted a wisdom tooth for an old lady who must have been certainly seventy-five years old. She had an upper set of false teeth. It showed but a small point at the surface, but I got out as perfect a tooth as I ever saw. The roots were straight and unusually long.

*Dr. Hitchcock.*—I would like to relate a peculiar case I once had of a wisdom tooth, which I came across in connection with Dr. Werner. The patient was a lady of about sixty-five years of age, who wore an artificial upper denture. The trouble began by a swelling away back in the mouth on the right upper side, followed by a discharge of pus. Examination with a probe resulted in finding, about three-fourths of an inch up, a hard substance, which moved, about readily, being surrounded by more or less dense bone. This substance, on being removed, was found to be a nodule, covered with enamel, evidently a partially-formed wisdom tooth.

*Dr. Stevens.*—I would like to say just one word, and that is with regard to extracting those lower wisdom teeth. I think if Dr. Brackett had an elevator similar to one which I have, he could have extracted the tooth of which he spoke without breaking it off, or without being obliged to put the patient under the influence of ether. An examination will show the wisdom tooth to hook backward, and therefore the force to be used in extraction should be applied in a way to unhook it. The elevator that I use is an ordinary elevator, so small as to be carried down between the tooth and the socket,—not using the twelve-year molar as a fulcrum at all, but using ordinary force in pressing it down, and then lifting it and turning it backward to unhook the tooth, and in this way they can be taken out remarkably easy. It is called the Coolidge elevator. I think Dr. Andrews has one. It is made with a large and long handle, and is so well adapted for extracting the wisdom tooth that I seldom use the forceps.

*Dr. Banfield.*—I would like to ask Dr. Brackett if he has noticed any disturbance of the wisdom teeth similar to that of a patient of mine? A young man of about twenty-four years came to my office, saying that he had been confined to his bed for nearly two weeks on account of supposed disturbance of his wisdom tooth. An examination found his teeth in good condition, but around the right inferior wisdom tooth the gum was considerably inflamed, which he said had given him much trouble; but the most peculiar feature was the dark-red color of the free margin of his gums, for one-eighth of an inch all around on both the upper and lower jaws. I have recently had a few cases of disturbance of the wisdom teeth, complicated with severe gingivitis, that made its appearance at the same time the patient was afflicted with la grippe.

*President Seabury.*—The next business is "Incidents of Practice and Presentation of Specimens."

Dr. E. G. Tucker exhibited a case of crown- and bridge-work that he inserted in a lady's mouth in 1844, and which was worn with satisfaction forty-five years, until 1889. The teeth were porcelain, carved, single teeth (left upper central incisor and right and left upper canines), set on gold plate, with gold pivots attached, and the three gold pivots were inserted into the hickory pivots which had previously been placed in the roots.

*Dr. Meriam.*—Mr. President, I present here some fine platinum and iridium tubes. I had them made some twelve or more years ago, and showed them at some of the society meetings. I do not think their value was understood at that time, for they received but little attention. The number of small syringe-points that are used now make me think that they are worth speaking of again. I have them here on a card just as they came back from the Minneapolis exhibit of 1889. They are useful for hollow posts and in making pieces for regulating, and for fine syringe-points. The smallest size is smaller than the finest tube now offered for sale; it telescopes into and can then be hard-soldered into the next larger size, this into the next larger, and so on, allowing for the making of a great variety of points. A foot of this tubing costs but a trifle. I gave my directions for making to Mr. George A. Warren, No. 7 Tremont Row, Boston. I did not, in the language of the trade journals, suggest them to him, and any one who wishes can order them made by him. It would be better, perhaps, for those at a distance to find some good workman, and after instructing him in their making, report his name to the dental societies in the vicinity, so that a greater number of practitioners will know how easy it is to obtain them.

*Dr. Williams.*—I have had several of them made, and I have found them very useful.

William H. Potter, D.M.D.,
*Editor American Academy Dental Science.*

